# Supplementary figures and images for: Hyperfunction of post-synaptic density protein 95 promotes seizure response in early-stage aβ pathology
Source: EMBO Rep. 2024 Feb 27;25(3):19. doi: 10.1038/s44319-024-00090-0 (PMC10933348; doi:10.1038/s44319-024-00090-0)

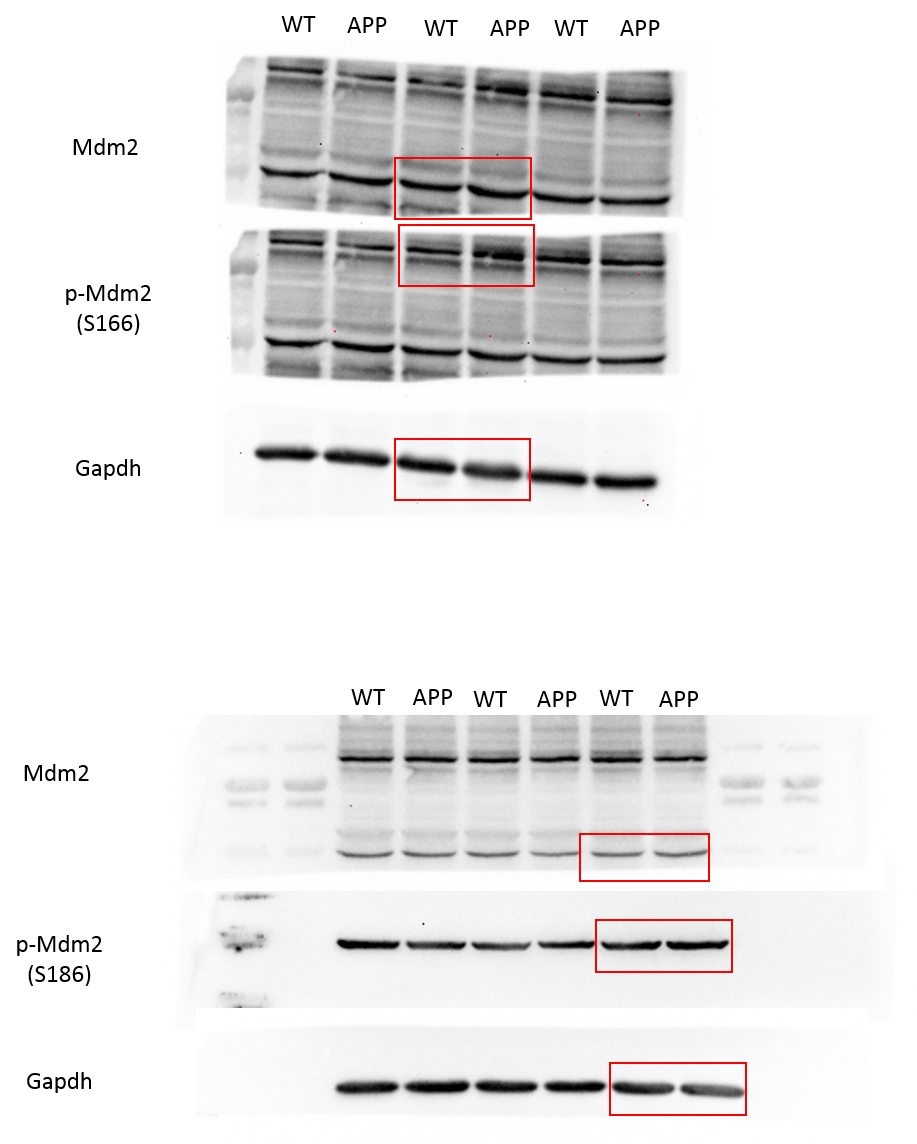

Supplement: Supplementary file 3 — Source Data Fig. 2 [file 44319_2024_90_MOESM3_ESM.zip › Figure 2 Source Data/2A/2A.tif]

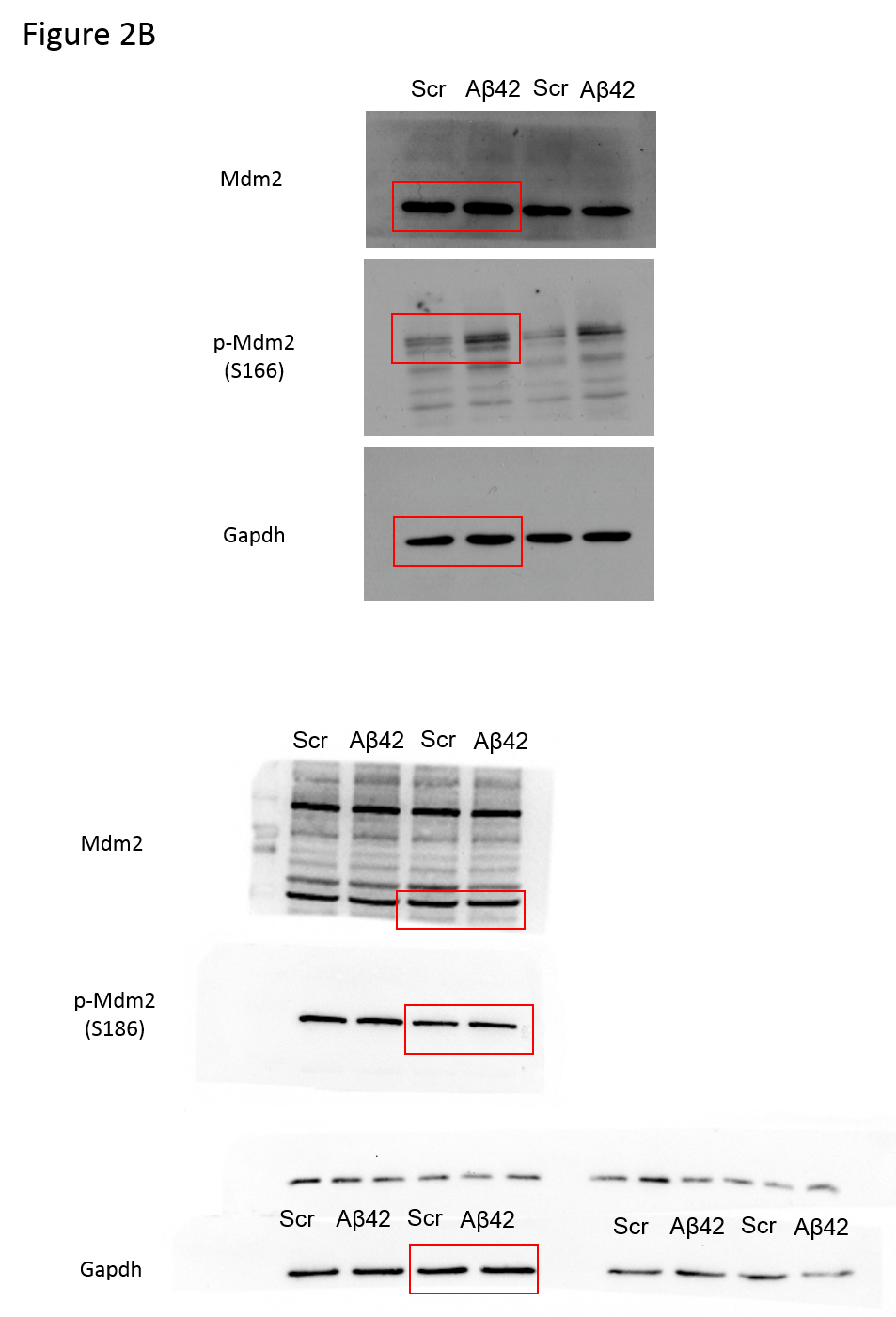

Supplement: Supplementary file 3 — Source Data Fig. 2 [file 44319_2024_90_MOESM3_ESM.zip › Figure 2 Source Data/2B/2B.tif]

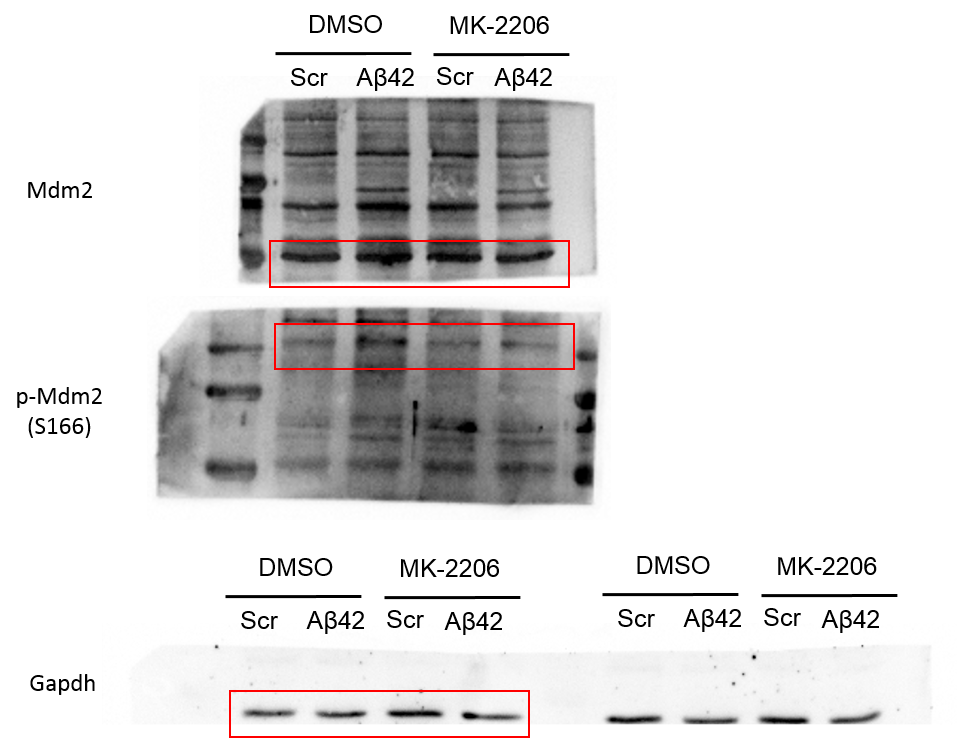

Supplement: Supplementary file 3 — Source Data Fig. 2 [file 44319_2024_90_MOESM3_ESM.zip › Figure 2 Source Data/2C/2C.tif]

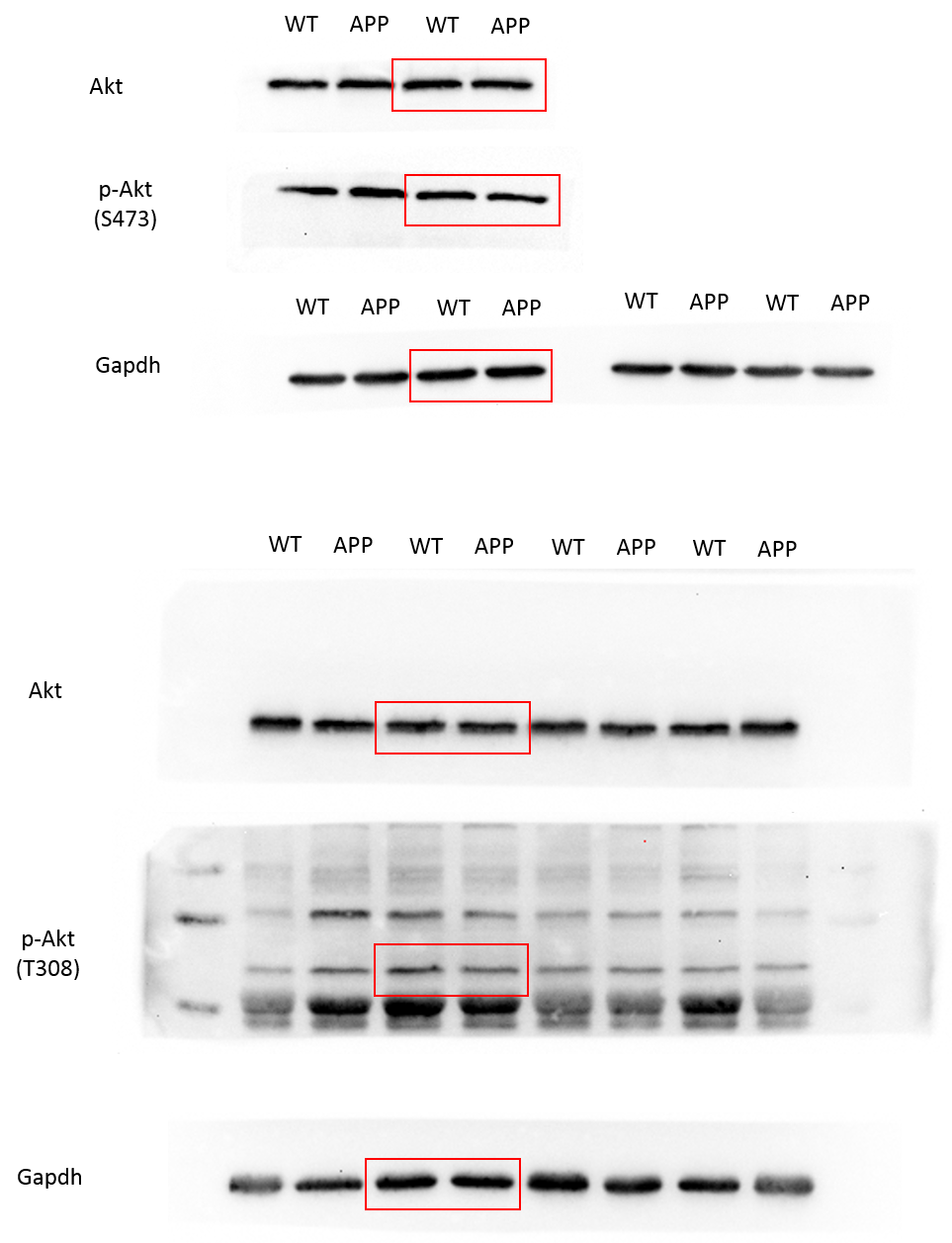

Supplement: Supplementary file 3 — Source Data Fig. 2 [file 44319_2024_90_MOESM3_ESM.zip › Figure 2 Source Data/2D/2D.tif]

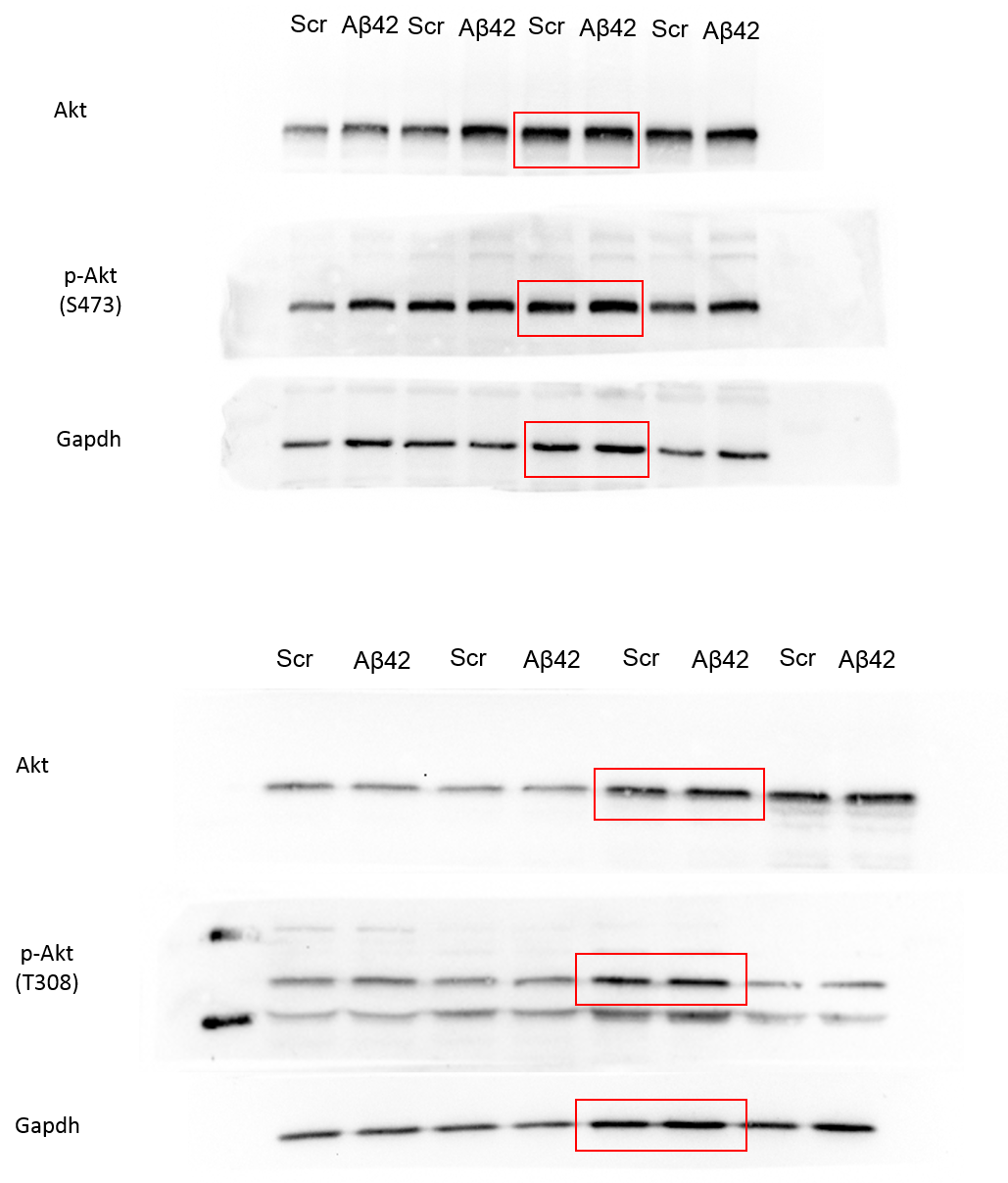

Supplement: Supplementary file 3 — Source Data Fig. 2 [file 44319_2024_90_MOESM3_ESM.zip › Figure 2 Source Data/2E/2E.tif]

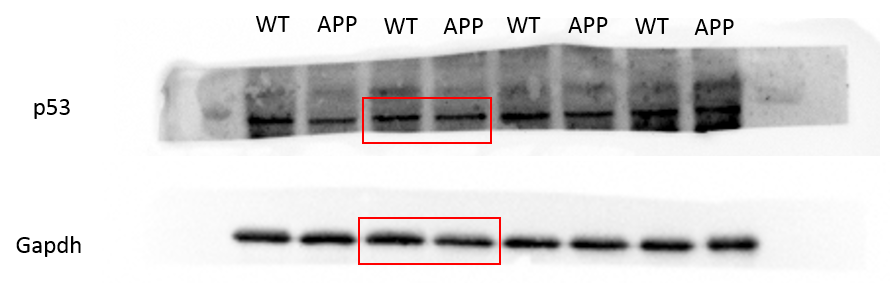

Supplement: Supplementary file 4 — Source Data Fig. 3 [file 44319_2024_90_MOESM4_ESM.zip › Figure 3 Source Data/3A/3A.tif]

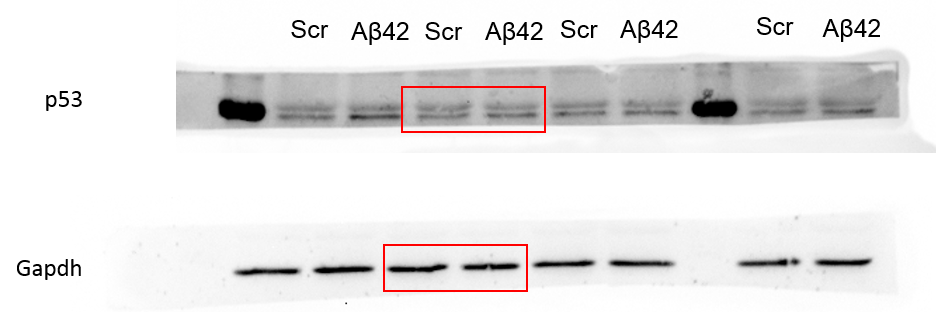

Supplement: Supplementary file 4 — Source Data Fig. 3 [file 44319_2024_90_MOESM4_ESM.zip › Figure 3 Source Data/3B/3B.tif]

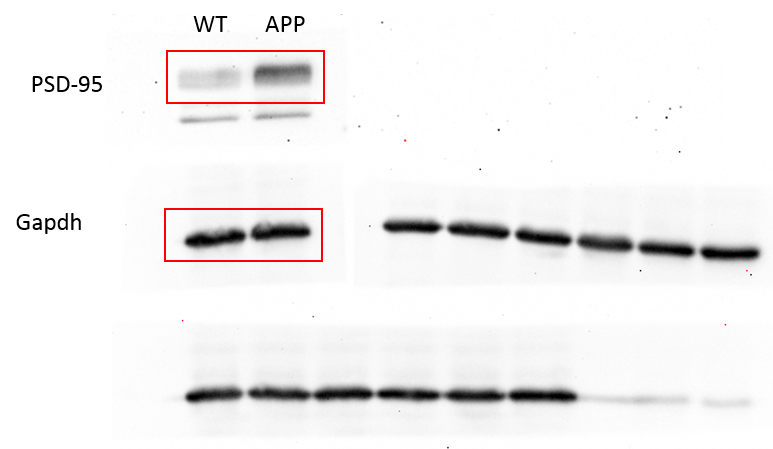

Supplement: Supplementary file 4 — Source Data Fig. 3 [file 44319_2024_90_MOESM4_ESM.zip › Figure 3 Source Data/3C/3C.tif]

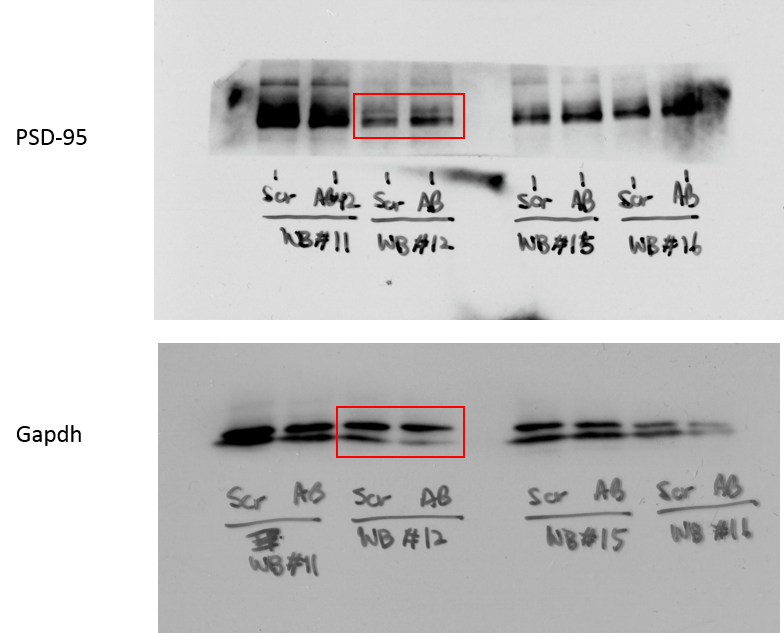

Supplement: Supplementary file 4 — Source Data Fig. 3 [file 44319_2024_90_MOESM4_ESM.zip › Figure 3 Source Data/3D/3D.tif]

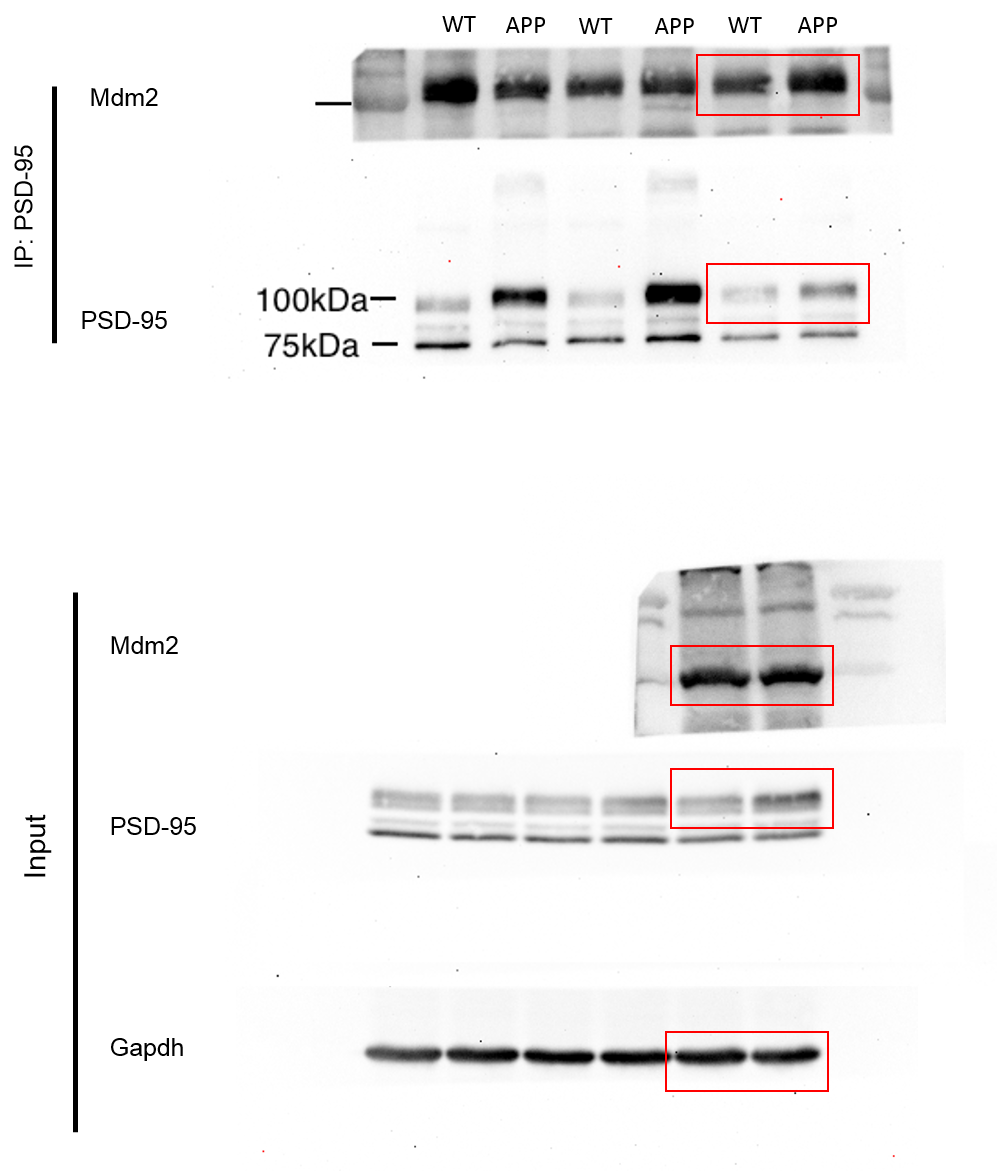

Supplement: Supplementary file 4 — Source Data Fig. 3 [file 44319_2024_90_MOESM4_ESM.zip › Figure 3 Source Data/3E/3E.tif]

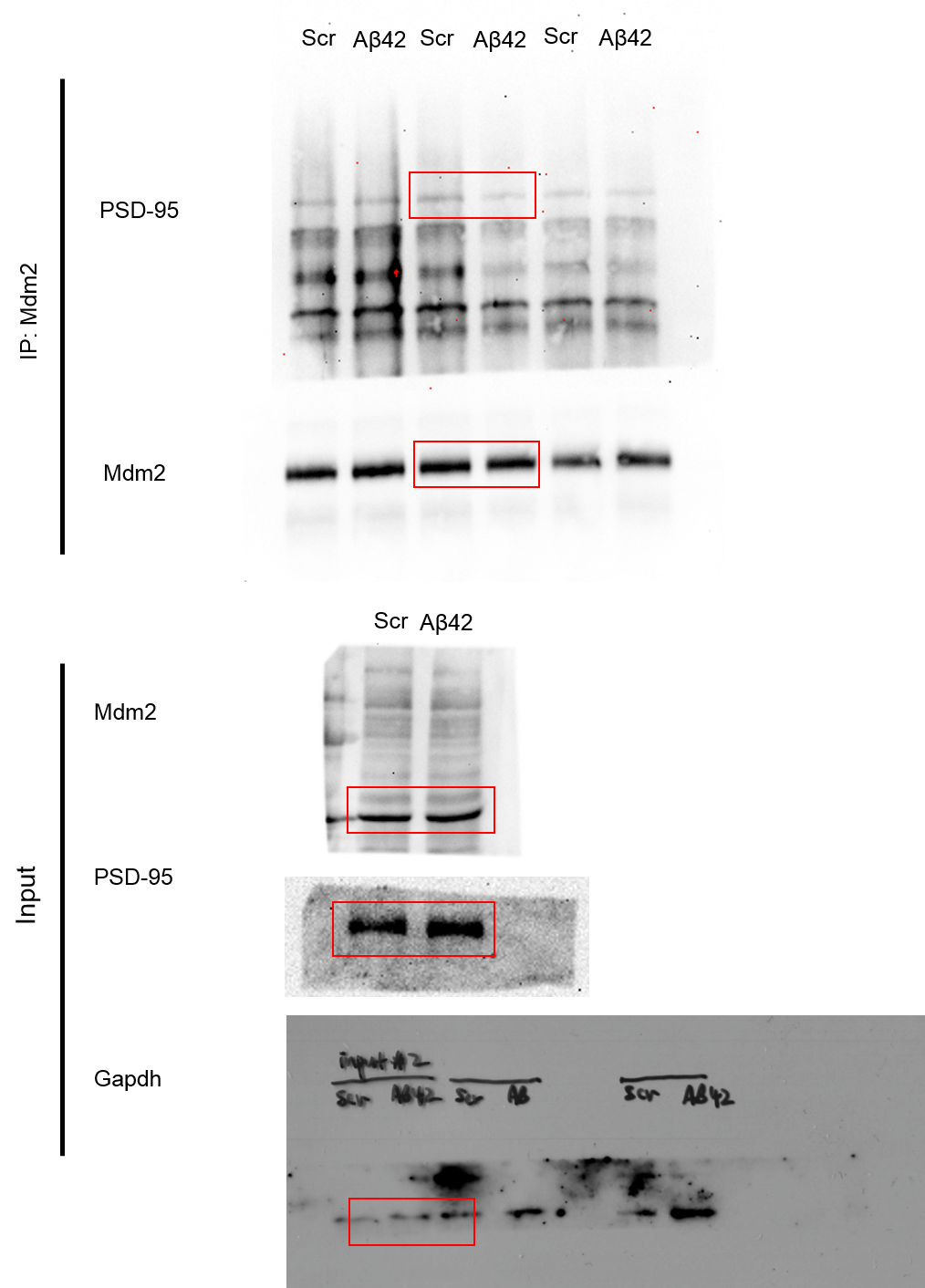

Supplement: Supplementary file 4 — Source Data Fig. 3 [file 44319_2024_90_MOESM4_ESM.zip › Figure 3 Source Data/3F/3F.tif]

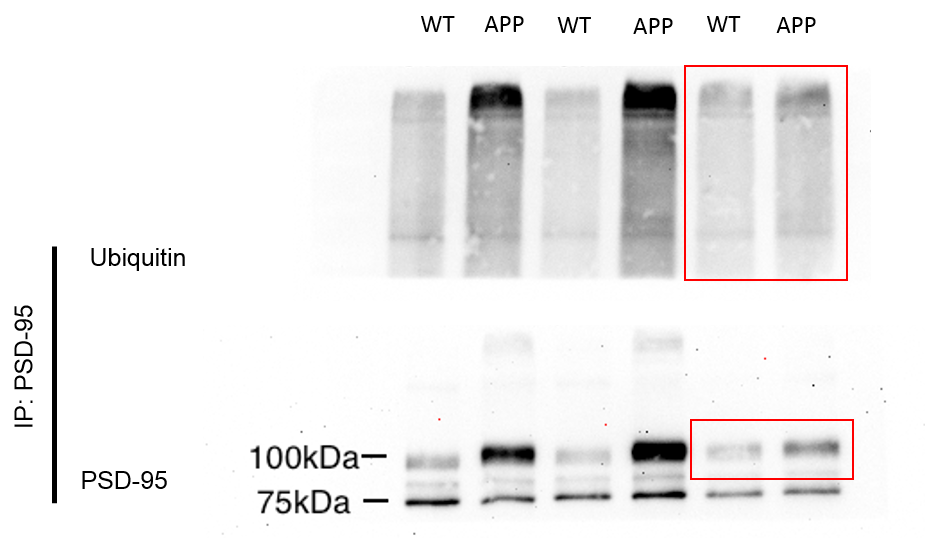

Supplement: Supplementary file 4 — Source Data Fig. 3 [file 44319_2024_90_MOESM4_ESM.zip › Figure 3 Source Data/3G/3G.tif]

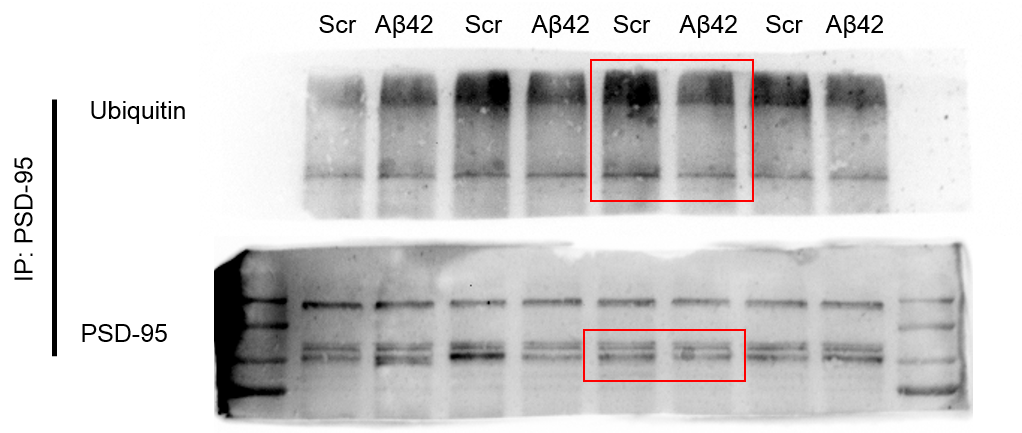

Supplement: Supplementary file 4 — Source Data Fig. 3 [file 44319_2024_90_MOESM4_ESM.zip › Figure 3 Source Data/3H/3H.tif]

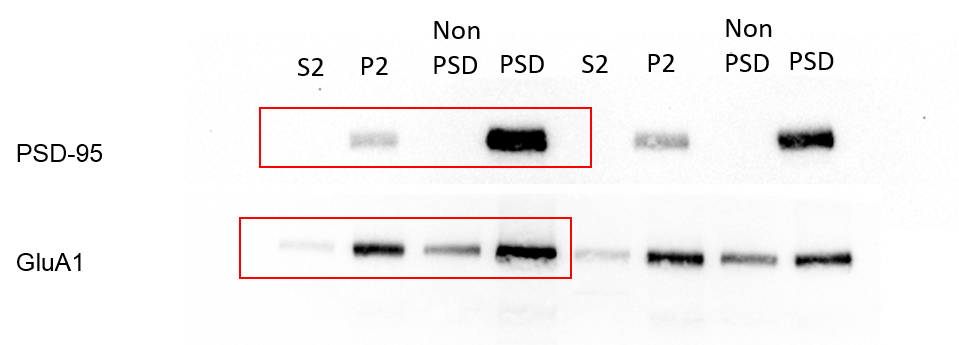

Supplement: Supplementary file 5 — Source Data Fig. 4 [file 44319_2024_90_MOESM5_ESM.zip › Figure 4 Source Data/4A/4A.tif]

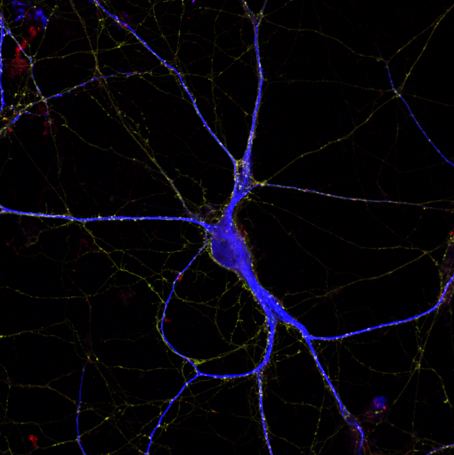

Supplement: Supplementary file 6 — Source Data Fig. 5 [file 44319_2024_90_MOESM6_ESM.zip › Figure 5 Source Data/5A/5A_WT_Ab.tif]

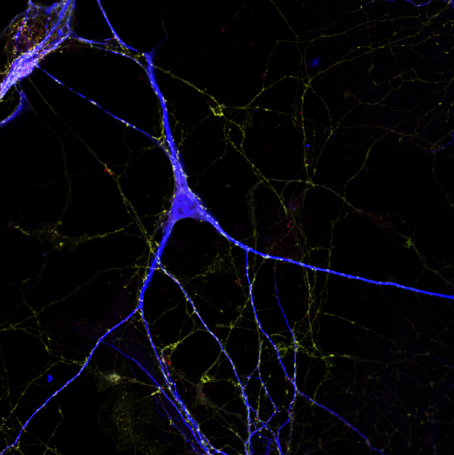

Supplement: Supplementary file 6 — Source Data Fig. 5 [file 44319_2024_90_MOESM6_ESM.zip › Figure 5 Source Data/5A/5A_WT_Scr.tif]

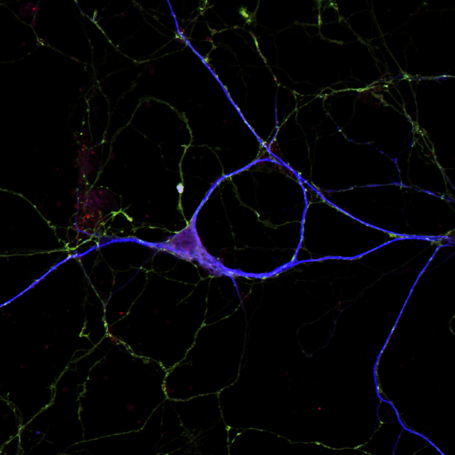

Supplement: Supplementary file 6 — Source Data Fig. 5 [file 44319_2024_90_MOESM6_ESM.zip › Figure 5 Source Data/5B/5B_PSD Het_Ab.tif]

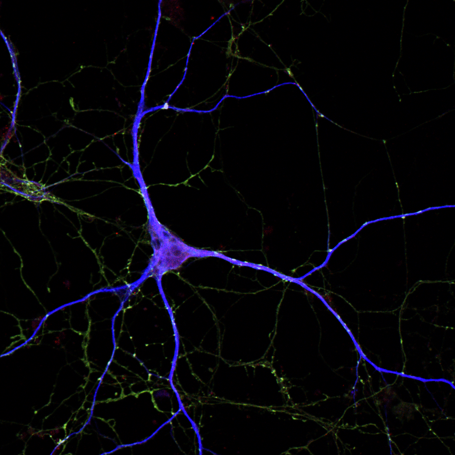

Supplement: Supplementary file 6 — Source Data Fig. 5 [file 44319_2024_90_MOESM6_ESM.zip › Figure 5 Source Data/5B/5B_PSD Het_Scr.tif]

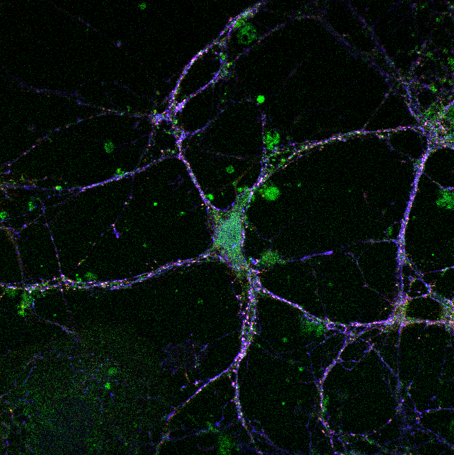

Supplement: Supplementary file 6 — Source Data Fig. 5 [file 44319_2024_90_MOESM6_ESM.zip › Figure 5 Source Data/5C/5C_KO_Ab.tif]

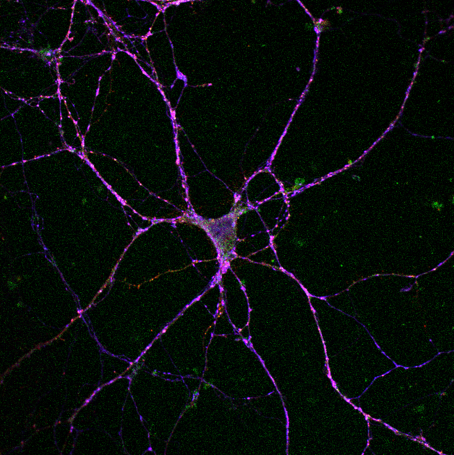

Supplement: Supplementary file 6 — Source Data Fig. 5 [file 44319_2024_90_MOESM6_ESM.zip › Figure 5 Source Data/5C/5C_KO_Scr.tif]

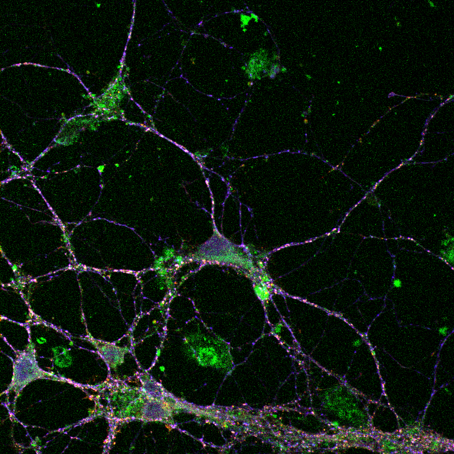

Supplement: Supplementary file 6 — Source Data Fig. 5 [file 44319_2024_90_MOESM6_ESM.zip › Figure 5 Source Data/5D/5D_KO_PSD_Ab.tif]

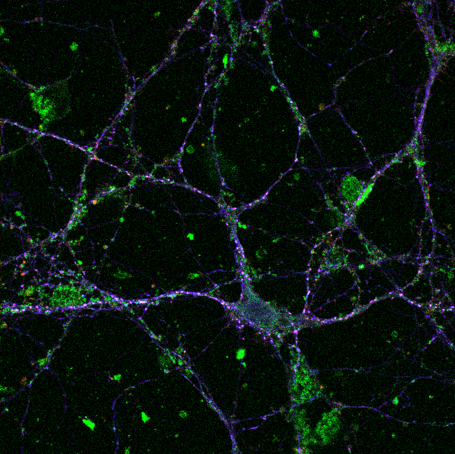

Supplement: Supplementary file 6 — Source Data Fig. 5 [file 44319_2024_90_MOESM6_ESM.zip › Figure 5 Source Data/5D/5D_KO_PSD_Scr.tif]

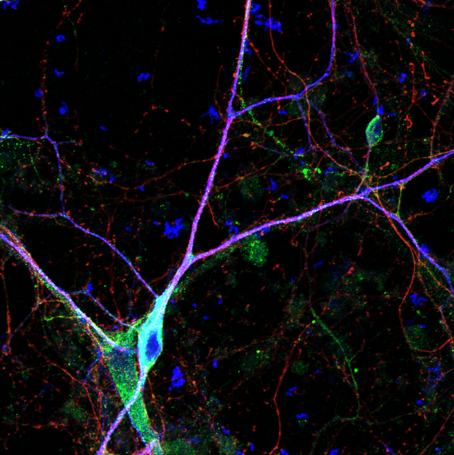

Supplement: Supplementary file 7 — Source Data Fig. 6 [file 44319_2024_90_MOESM7_ESM.zip › Figure 6 Source Data/6A/6A_WT_Ab_GluA1.tif]

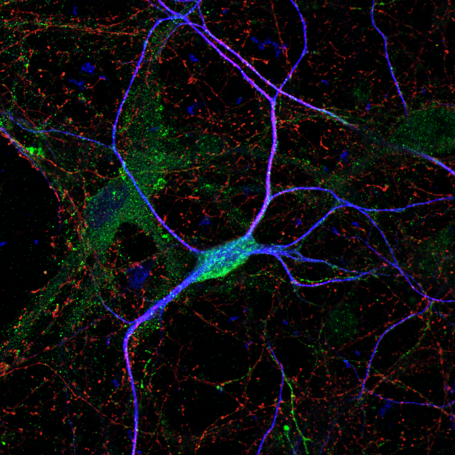

Supplement: Supplementary file 7 — Source Data Fig. 6 [file 44319_2024_90_MOESM7_ESM.zip › Figure 6 Source Data/6A/6A_WT_Scr_GluA1.tif]

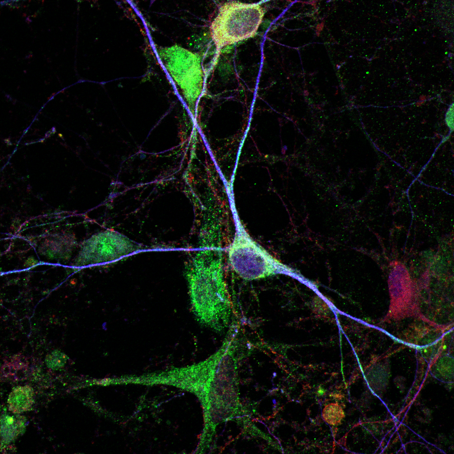

Supplement: Supplementary file 7 — Source Data Fig. 6 [file 44319_2024_90_MOESM7_ESM.zip › Figure 6 Source Data/6B/6B_WT_Ab_GluA2.tif]

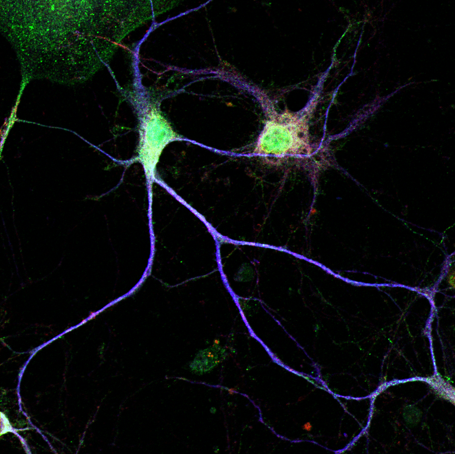

Supplement: Supplementary file 7 — Source Data Fig. 6 [file 44319_2024_90_MOESM7_ESM.zip › Figure 6 Source Data/6B/6B_WT_Scr_GluA2.tif]

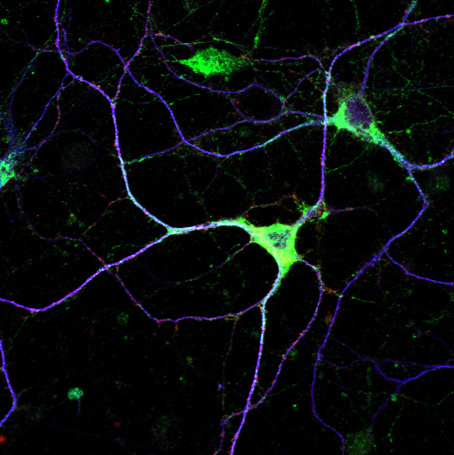

Supplement: Supplementary file 7 — Source Data Fig. 6 [file 44319_2024_90_MOESM7_ESM.zip › Figure 6 Source Data/6C/6C_Het_Ab_GluA1.tif]

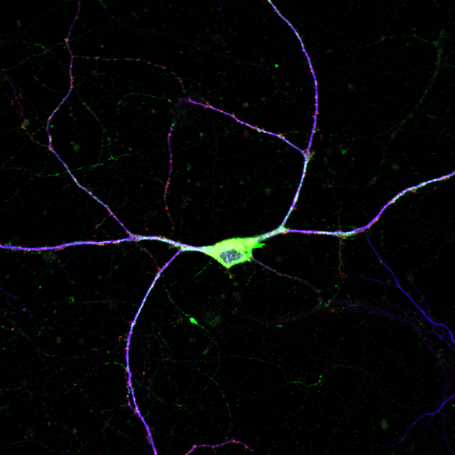

Supplement: Supplementary file 7 — Source Data Fig. 6 [file 44319_2024_90_MOESM7_ESM.zip › Figure 6 Source Data/6C/6C_Het_Scr_GluA1.tif]

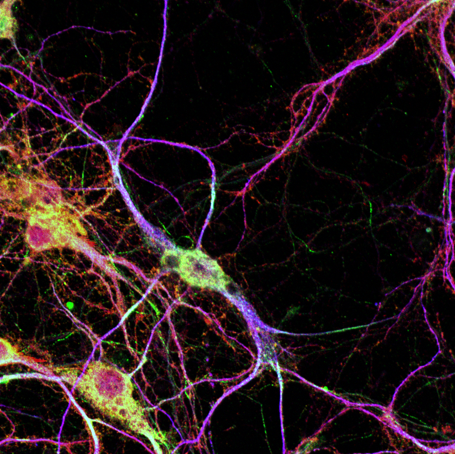

Supplement: Supplementary file 7 — Source Data Fig. 6 [file 44319_2024_90_MOESM7_ESM.zip › Figure 6 Source Data/6D/6D_Het_Ab_GluA2.tif]

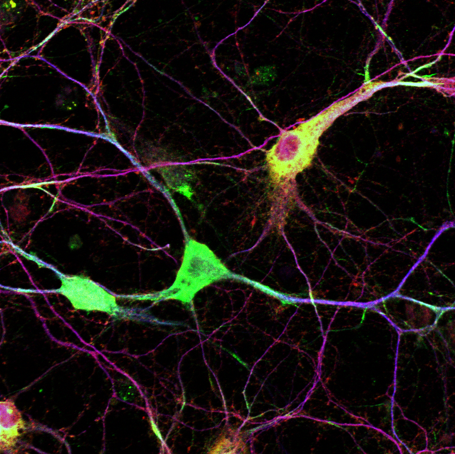

Supplement: Supplementary file 7 — Source Data Fig. 6 [file 44319_2024_90_MOESM7_ESM.zip › Figure 6 Source Data/6D/6D_Het_Scr_GluA2.tif]

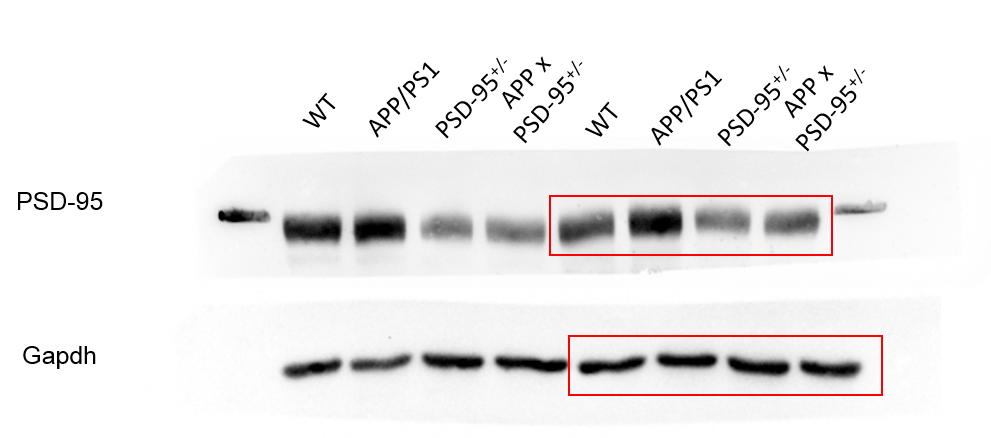

Supplement: Supplementary file 8 — Source Data Fig. 7 [file 44319_2024_90_MOESM8_ESM.zip › Figure 7 Source Data/7A/7A.tif]

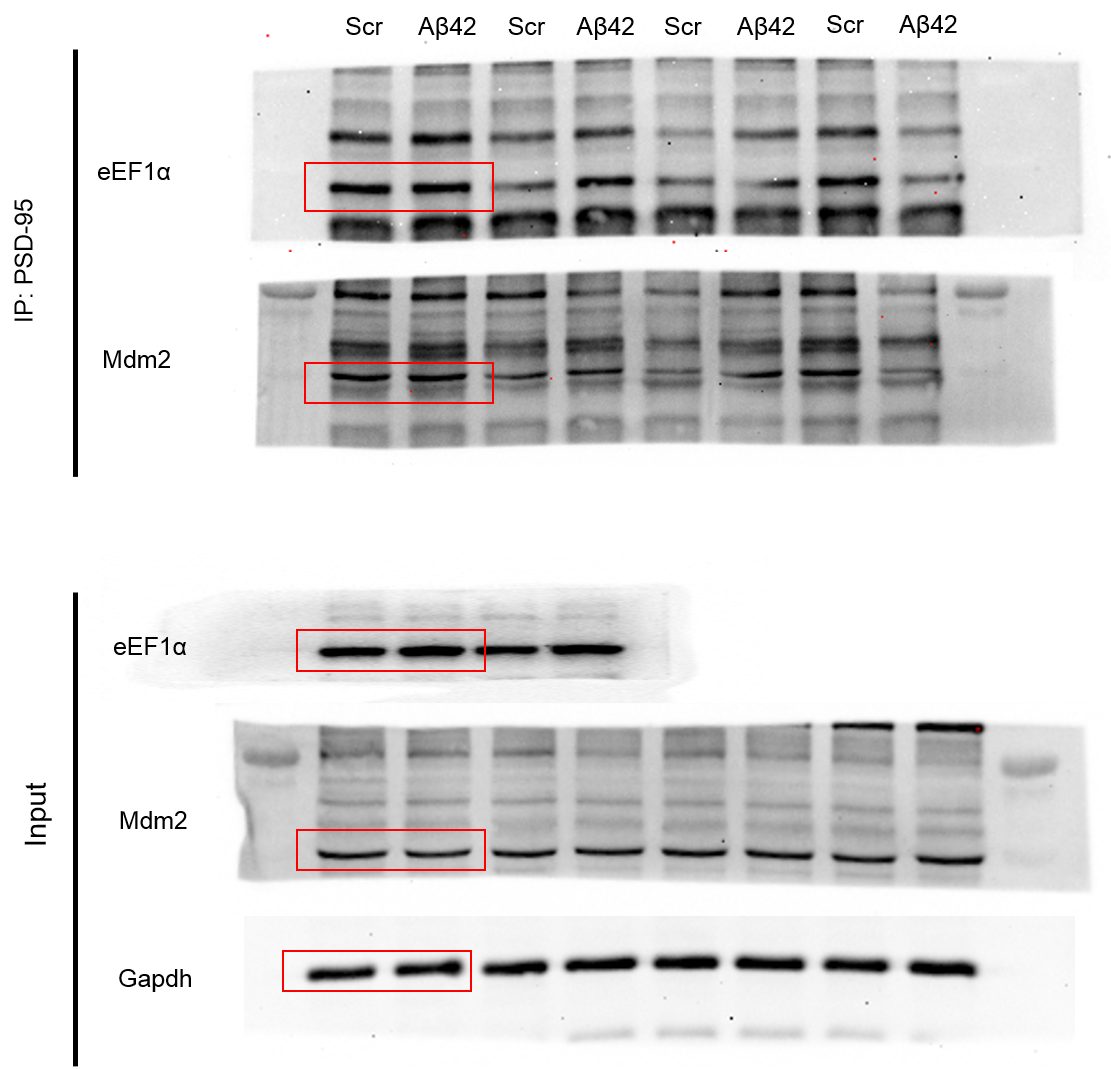

Supplement: Supplementary file 9 — Source Data EVs Figures [file 44319_2024_90_MOESM9_ESM.zip › Figure EV2 Source Data/EV2.tif]

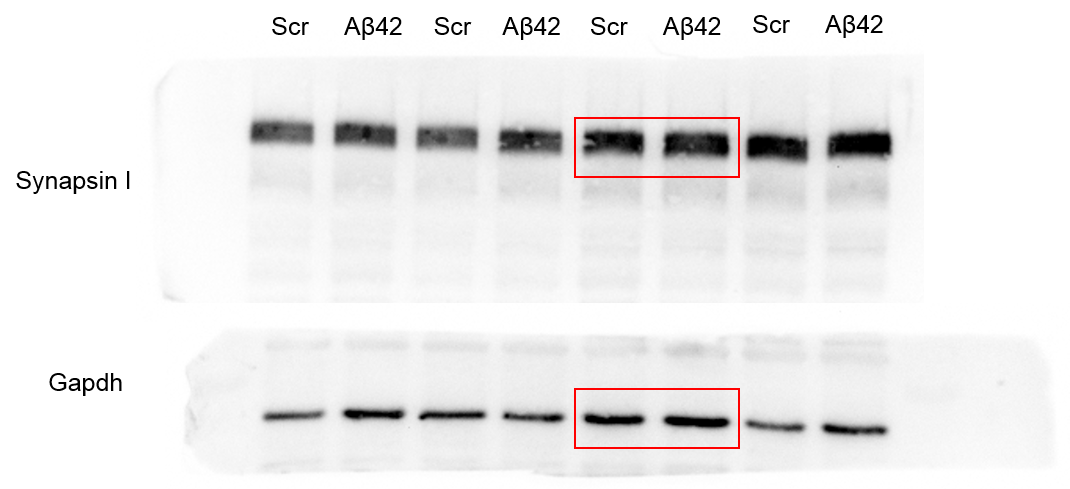

Supplement: Supplementary file 9 — Source Data EVs Figures [file 44319_2024_90_MOESM9_ESM.zip › Figure EV3 Source Data/EV3.tif]
