# Supplementary material for: Hyperfunction of post-synaptic density protein 95 promotes seizure response in early-stage aβ pathology
Source: EMBO Rep. 2024 Feb 27;25(3):19. doi: 10.1038/s44319-024-00090-0 (PMC10933348; doi:10.1038/s44319-024-00090-0)
Supplement: Supplementary file 11 — Expanded View Figures [file 44319_2024_90_MOESM11_ESM.pdf]

Expanded View Figures

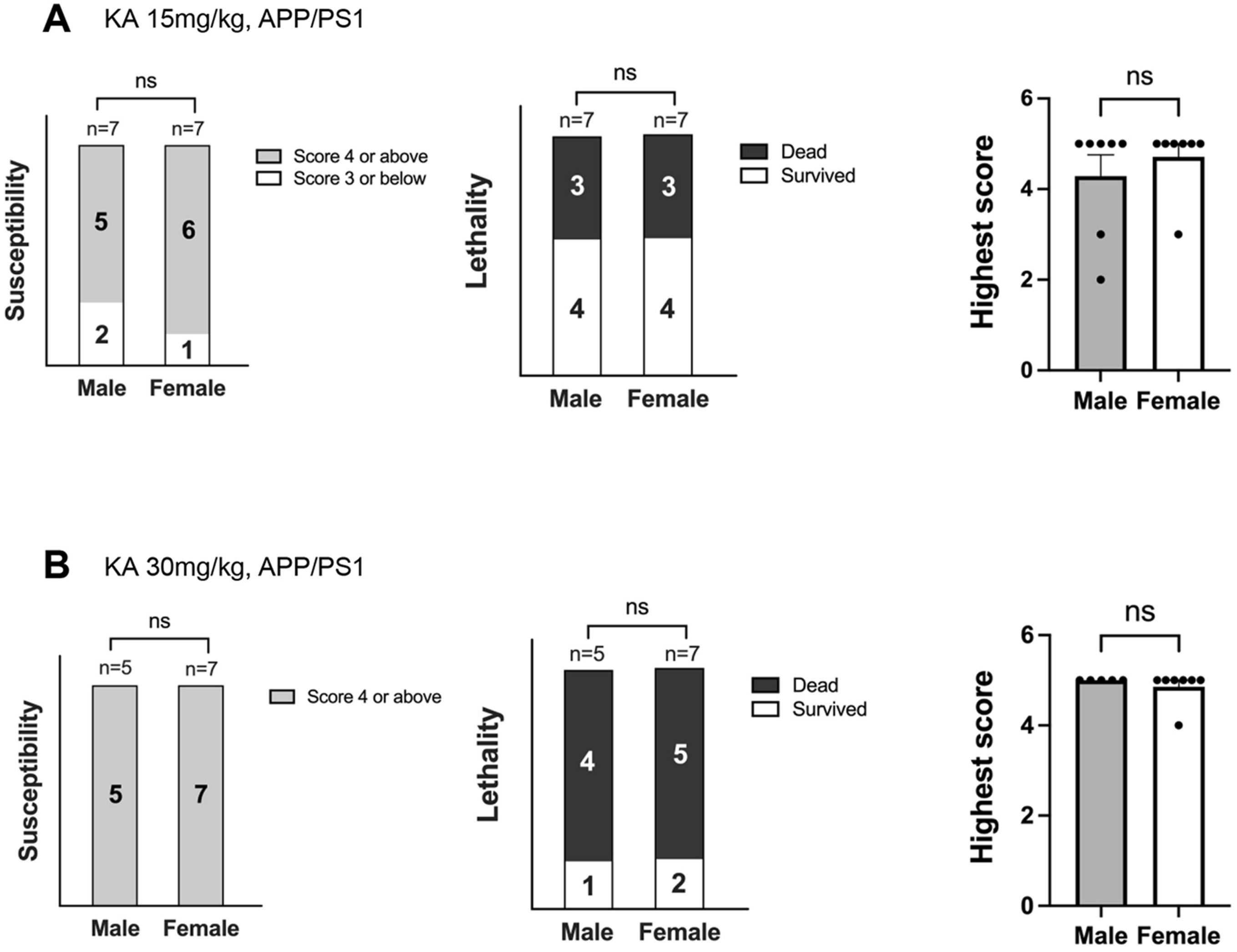

Figure EV1. Seizure severity in young APP/PS1 does not exhibit sex differences.

(**A**, **B**) Quantification of seizure susceptibility, lethality, and highest score in male and female APP/PS1 mice. Two different doses of 15 mg/kg (**A**) and 30 mg/kg (**B**) were intraperitoneally injected into mice. Significance was determined by Fisher's exact test (seizure susceptibility; $P > 0.9999$ for both 15 and 30 mg/kg and lethality; $P > 0.9999$ for both 15 and 30 mg/kg) or Mann–Whitney U test (highest score; $P = 0.7308$ for 15 mg/kg and $P > 0.9999$ for 30 mg/kg). Data are represented as mean ± SEM with ns: non-significant. Source data are available online for this figure.

 © The Author(s)

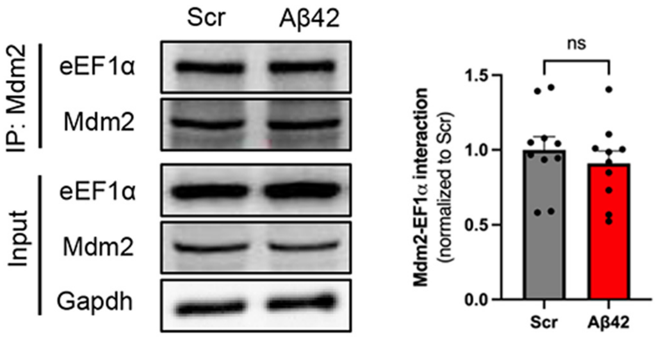

Figure EV2. A$\beta_{1\text{-}42}$ does not alter the interaction between eEF1α and Mdm2.

Quantification of interaction between eEF1α and Mdm2 after co-immunoprecipitation and representative western blots using lysates from WT primary cortical neuron cultures treated with A$\beta_{1\text{-}42}$ (Aβ42, 1 μM) or scrambled Aβ peptide (Scr, 1 μM) for 2 h at DIV 12–14. $n = 10$ at least from three independent cultures for both Scr and Aβ42 groups. Significance was determined by Student's t test ($P = 0.4605$). Data are represented as mean ± SEM with ns: non-significant. Source data are available online for this figure.

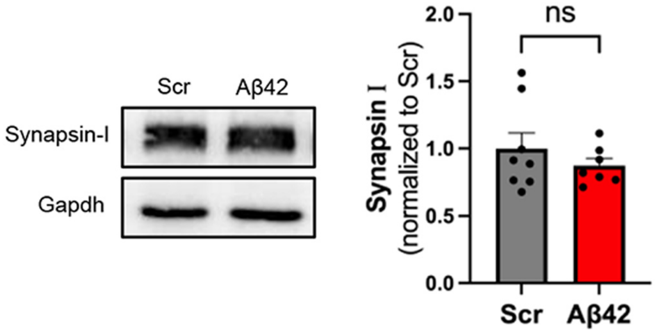

Figure EV3. $A\beta_{1\text{-}42}$ does not promote the total level of Synapsin-I.

Quantification of Synapsin-I and representative western blots from WT primary cortical neuron cultures treated with amyloid-beta 1–42 (Aβ42; 1 μM) or scrambled Aβ peptide (Scr, 1 μM) for 2 h at DIV 12–14. $n = 8$ and 7 for Scr and Aβ42 groups, respectively. Significance was determined by Student's t test ($P = 0.3604$). Data are represented as mean ± SEM with ns: non-significant. Source data are available online for this figure.

 © The Author(s)

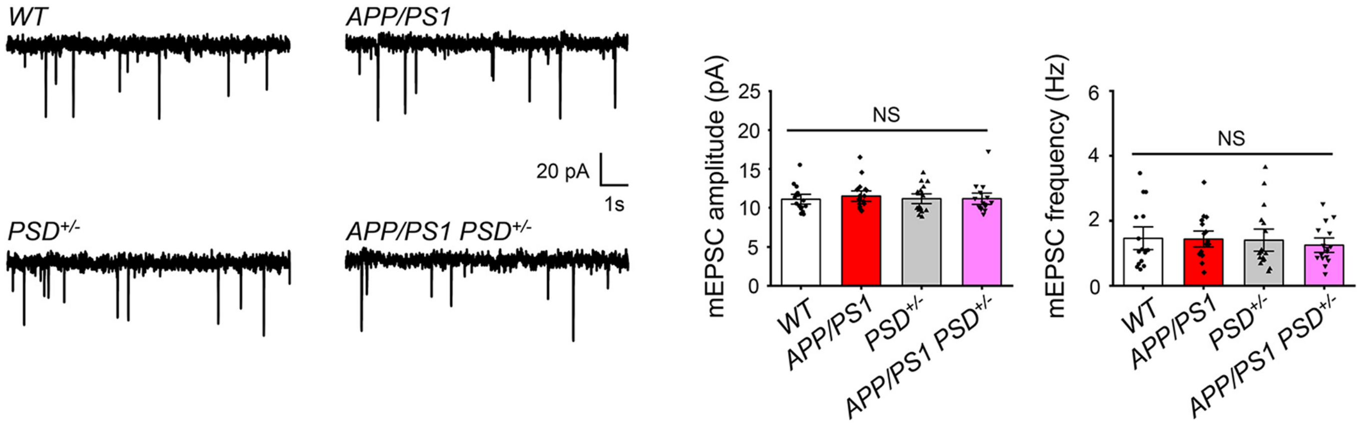

Figure EV4. No changes in mEPSCs were observed in hippocampal CA1 neurons in PSD-95$^{-/+}$ or APP/PS1 mice.

Voltage-clamp recordings of mEPSCs from CA1 pyramidal cells in acute hippocampal slice of WT ($n = 16$ cells from 6 mice), APP/PS1 ($n = 17$ cells from 7 mice), PSD$^{-/+}$ ($n = 17$ cells from 6 mice), and APP/PS1 PSD$^{-/+}$ (16 cells from 6 mice) mice. Holding potential was −70 mV. Representative mEPSC traces (left) and quantification of mEPSC amplitude and frequency (right) are shown. Data were analyzed by one-way ANOVA with Tukey test and presented as mean ± SEM with NS: non-significant. Source data are available online for this figure.

© The Author(s)
